# *In Vivo* Processing of DNase Colicins E2 and E7 Is Required for Their Import into the Cytoplasm of Target Cells

**DOI:** 10.1371/journal.pone.0096549

**Published:** 2014-05-19

**Authors:** Liliana Mora, Miklos de Zamaroczy

**Affiliations:** Institut de Biologie Physico-Chimique, CNRS, UPR 9073, Paris, France; Centre National de la Recherche Scientifique, Aix-Marseille Université, France

## Abstract

DNase colicins E2 and E7, both of which appropriate the BtuB/Tol translocation machinery to cross the outer membrane, undergo a processing step as they enter the cytoplasm. This endoproteolytic cleavage is essential for their killing action. A processed form of the same size, 18.5 kDa, which corresponds to the C-terminal catalytic domain, was detected in the cytoplasm of bacteria treated with either of the two DNase colicins. The inner-membrane protease FtsH is necessary for the processing that allows the translocation of the colicin DNase domain into the cytoplasm. The processing occurs near residue D420, at the same position as the FtsH-dependent cleavage in RNase colicins E3 and D. The cleavage site is located 30 amino acids upstream of the DNase domain. In contrast, the previously reported periplasm-dependent colicin cleavage, located at R452 in colicin E2, was shown to be generated by the outer-membrane protease OmpT and we show that this cleavage is not physiologically relevant for colicin import. Residue R452, whose mutated derivatives led to toxicity defect, was shown to have no role in colicin processing and translocation, but it plays a key role in the catalytic activity, as previously reported for other DNase colicins. Membrane associated forms of colicins E2 and E7 were detected on target cells as proteinase K resistant peptides, which include both the receptor-binding and DNase domains. A similar, but much less proteinase K-resistant form was also detected with RNase colicin E3. These colicin forms are not relevant for colicin import, but their detection on the cell surface indicates that whole nuclease-colicin molecules are found in a stable association with the outer-membrane receptor BtuB of the target cells.

## Introduction

Under stress conditions the production of colicins is a response that *E. coli* strains have developed to facilitate the invasion of environmental populations competing for limited nutrients [1]. The antibacterial toxins, once released into the extracellular medium, selectively kill non-colicin producing strains, but they are not toxic against colicin producer cells [2]. Colicinogenic strains are protected against both endogenous and exogenous toxin molecules by the constitutive expression of an immunity (Imm) protein, which in the case of nuclease type colicins forms a tight heterodimer complex with the colicins [3], [4].

Nuclease colicins are one of the two main classes of colicins. Both require colicin translocation across the outer membrane. While killing by one class is based on a pore-forming activity at the inner membrane that results in depolarization of the target cell, the second utilizes nucleolytic activities targeting chromosomal DNA (colicins E2, E7 and E9), ribosomal (colicin E3) or transfer RNAs (colicins E5 and D). The nuclease type of colicins requires their translocation across both the outer and inner membranes. Most colicins have structurally identifiable N-terminal, central and C-terminal domains. During early steps of invasion the first two domains are required for receptor binding and translocation across the outer membrane. The E-type colicins appropriate the vitamin B12-uptake machinery of target cells to enable the C-terminal cell-killing catalytic domain to reach the periplasm [5]–[7]. Binding of the E-type nuclease colicin-Imm complexes to the cell surface receptor BtuB, leads to the dissociation of the Imm protein, which is released into the medium [8], [9]. The binding *per se* of the complex to the BtuB receptor is however not enough to liberate the Imm protein. The dissociation of colicins E9- and E2-Imm complexes requires the unfolding of the colicin, as it contacts the energy-transducing Tol system in the periplasm, and thus allowing the access to the inner-membrane-derived pmf (protonmotive force) [6].

We previously demonstrated that, in the case of two RNase colicins, D and E3, an endoproteolytic processing is essential to transfer the cytotoxic domain across the inner membrane. As a consequence, only the cytotoxic C-terminal domains were detected in the cytoplasm of colicin D or E3-exposed cells [10], [11]. During the last few years, the essential, inner-membrane signal-peptidase LepB was shown to be required specifically for the toxicity of colicin D, whereas the inner-membrane protease FtsH is required for the toxicity of all nuclease colicins [12], [13]. LepB interacts directly with the central domain of the tRNase colicin D *in vitro* and it was further shown that this peptidase is a specific but non-catalytic requirement for the processing of colicin D. FtsH was shown to be indispensable for the processing and/or translocation of RNase colicins [10].

Cleavage of DNase colicin E7 in the presence of periplasmic extracts was previously reported, but the catalytic enzyme was not identified [14], [15]. Analysis of point mutations introduced at the *in vitro* determined cleavage site of colicin E7 or E2 led to a decrease or loss of colicin toxicity and seemed to prevent the cleavage of the colicin *in vivo*, so that the authors claimed that the periplasmic cleavage was responsible for the colicin processing, required for colicin import and cell killing [15], [16].

The catalytic domains of DNase colicins exhibited non-voltage-gated channel-forming activity in planar lipid bilayers, which involved changes in their conformation [17]. Such channels, unlike those formed by pore-forming colicins, are not directly responsible for cell killing, but, as proposed for colicin E9, may allow “self-propulsion” of the colicin into the cytoplasm, driven by an electrostatic association of the DNase domain with the inner membrane [18]. Although an association between the RNase colicin E3 and anionic phospholipid surfaces has been reported, no RNase colicin has been found to exhibit any channel forming activity [19], [20].

In this work, we have identified the *in vivo* processed forms of two DNase colicins, E2 and E7, as the final colicin forms present in the cytoplasm of colicin-treated bacteria. As in the case of the RNase colicins, the endoproteolytic cleavage was shown to require the inner-membrane protease FtsH. The processing of DNase colicins does not occur if any of the earlier import steps are prevented by specific mutations affecting the receptor BtuB or the energy-transducing Tol system. This study allowed us to evaluate the processing and translocation of DNase colicins into the cytoplasm in comparison with those of RNase colicins.

## Materials and Methods

### Bacterial Strains and Plasmids

*E. coli* K12 strains C600 and JM101 were used as wild-type strains. DH5α and XL1- blue were used as the host strain for cloning and mutagenesis, respectively. All other strains and plasmids used or constructed are listed in Table 1.

**Table 1:** Bacterial strains and plasmids used in this study.

Strain or plasmid	Genetic description	References
**AD202**	*ompT*1000::*kan*, Km^R^	[48]
**BL21(DE3)**	Δ*ompT*	[49]
**JCL8789**	188 *nadA*::Tn*10*, Δ*tolB*-*pal*, Tet^R^	[50]
**JCL11650**	169 *btuB*::Tn*10*, Tet^R^	[50]
**JCL11619**	188 *nadA*::Tn*10*, Δ*tolA*, Tet^R^	[50]
**JW2203-1 (9781)**	Δ*ompC*768::kan, Km^R^	CGSC^a^
**JCL8928**	8056 zcb::Tn*10*, Δ*ompF*627, Tet^R^	[50]
**LM2013**	Δ*ompC*768-Δ*ompF*627, Tet^R^, Km^R^	This work
	(by P1 transduction from JW2203-1 to JCL8928)	
**AR3291/1734**	MG1655 *sfhC zad*::Tn*10*, *ftsH3*::kan, Tet^R^, Km^R^	[10], [51]
**p** ***ftsH***	pACYC184, *ftsH* (at *Sal*I/*Hin*DIII), Cm^R^	[10]
**pColE2-P9**	colicin E2 *cea-cei-cel* b	[52]
**pColE2I**	pACDuet-1, *cea*-*cei*(tag-His_6_), (at *Nde*I/*Bam*HI), Cm^R^	This work
**pK317**	colicin E7 *cea-cei-cel* b	[53]
**pColE7I**	pET11a, *cea*-*cei*(tag-His_6_), (at *Nde*I/*Bam*HI), Amp^R^	This work
**pColE3I**	pET11a, *cea*-*cei*(tag-His_6_) (at *Nde*I/*Bam*HI), Amp^R^	[10]
**pColE3/ColE2-ImmE2**	hybrid colicin: junction at ColE3 R454/ColE2 K457	This work
**pColE2/ColE3-ImmE3**	hybrid colicin: junction at ColE2 G456/ColE3 K455	This work

a The Coli Genetic Stock Center.

b *cea, cei, cel* are structural genes for colicin E encoding toxin, immunity and lysis proteins.

### Cloning and Purification of Colicins - Antiserum Production

Plasmids carrying the *cea* and *cei* genes (with a C-terminal His_6_ tag) of the colicin E2 or E7 operon (Table 1), were constructed by PCR from pColE2-P9 and pK317 template DNAs (Table 1), respectively, with oligonucleotides introducing *Nde*I restriction sites upstream (primer “Nt-ColE2 or “Nt-ColE7”) and *Bam*HI sites downstream (primer “Ct-ImmE2” or “Ct-ImmE7”; Table 2) of amplified regions. The amplified double stranded DNAs were inserted after *Nde*I-*Bam*HI digestion, into the equivalent restriction sites of the pACDuet-1 or pET11a vector, to yield plasmids pColE2I and pColE7I (Table 1), respectively.

**Table 2:** Oligonucleotide primers designed and used for wild-type or hybrid colicin cloning.

Primer name	Sequence 5′-3′	Function	Source
**Nt-ColE7**	GGAATTC***CATATG***AGCGGTGGaGATGGAC	ColE7 sense cp^a^	Tw^b^
**Ct-ImmE7**	TCGCTC**G** ***GATCC*** TCAGTGGTGGTGGTGGTGGTGGCCCTGTTTAAATCCTGGCTTAC	ColE7 antisense cp	Tw
**Nt-ColE2**	GGAATTC***CATATG***AGCGGTGGcGATGGAC	ColE2 sense cp	Tw
**Ct-ImmE2**	TCGCTC***GGATCC*** TCAGTGGTGGTGGTGGTGGTGGCCCTGTTTAAATCCTGACTTACCGTTAG	ColE2 antisense cp	Tw
**Nt-ColE3**	GGAATTC***CATATG***AGCGGTGGCGATGGACGCGGCCATAACACG	ColE3 sense cp	Tw
**Ct-ImmE3**	TCGC***GGATCC***ATTAGTGGTGGTGGTGGTGGTGCCAATCACCATCACGATAATCAAACGAAAC	ColE3 antisense cp	Tw
**ColE2-G456**	CTTCCCTGGCTTATTCCGTTTACTCTCC	ColE2 antisense p G456	Tw
**ColE3-R454**	TTTTCTGGGCTTATTCTTTTCATCG	ColE3 antisense p R454	Tw
**ColE3-ColE2**	GGAGTGCTGAAAATAATTTAAACGATGAAAAGAATAAGCCCAGAAAAGCGACAGGTAAAGGTAAACCAGTTG	Jonction sense pColE3-R440-R454/ColE2- K457-V465	Tw
**ColE2-ColE3**	CTAAGGATAAATTAGATAAGGAGAGTAAACGGAATAAGCCAGGGAAGGGTTTTAAAGATTACGGGCATGATTATCATCC	Jonction sense pColE2-A442-G456/ColE3-K455-H465	Tw

a Cloning primer (*Nde*I or *Bam*H1 restriction sites are in bold italic; start or stop codons are double underlined; C-terminal 6His-tag is underlined).

b This work.

Unlabelled colicin E2 (or labelled *in vivo* with [^35^S]Met; 1000 Ci/mmol, Amersham) in complex with its immunity protein (ImmE2) carrying a C-terminal His_6_ tag was purified by affinity chromatography, as previously described for RNase colicins [10], [12], [21], from BL21(DE3) bacteria carrying the plasmid pColE2I. Similarly, colicin E7-ImmE7(Ct-His_6_) was purified from BL21(DE3) bacteria, carrying the plasmid pColE7I. His-tagged colicin-Imm complexes were denaturated in 9 M urea, the His-tagged ImmE2 (or ImmE7) was retained by two passages over Ni-NTA column. Colicin E2 (or colicin E7) freed of its Imm protein present in the “flow through” was concentrated (ultrafiltration Amicon Ultra-4 10K NMWL; Millipore) after dialysis. Purified colicin-Imm complexes were used for specific polyclonal antiserum production against colicin E2-ImmE2 or colicin E7-ImmE7. Antiserums anti-colicin E2 and E7 cross react because of the similar R- and T- and DNase domains of these colicins. Antiserums anti-colicin E2-ImmE2 and the previously produced anti-colicin E3-ImmE3 [10], [12], [21] cross react with the highly conserved receptor-binding (R-) and translocation (T-) domains of each colicin E2 or E3, but they recognize specifically the C-terminal catalytic domain of the colicin used for their production.

### Construction of Chimeric Colicin E Variants

Three-step PCR amplification technique was used to create hybrid colicin E3/colicin E2-ImmE2, by employing plasmids pColE3I and pColE2I (Table 1) as templates, for the first two PCR, respectively. To create this chimeric variant (fused at position R454 in colicin E3) the first PCR used an upstream oligonucleotide “Nt-ColE3” (Table 2) (containing the *Nde*I restriction site that includes an ATG codon, followed by nucleotides of the *cda* coding sequence of colicin E3 starting from its second codon) and a downstream oligonucleotide “ColE3-R454”, complementary to *cda* and starting at R454 (Table 2). The second PCR amplification used an upstream oligonucleotide “ColE3-ColE2” (Table 2) that is in part complementary to the downstream oligonucleotide used in the first PCR and is in-frame with codons starting at K457 of colicin E2, together with a downstream oligonucleotide “Ct-ImmE2”, complementary to 6 His codons preceding the stop codon of *cei* (encoding ImmE2) and the *Bam*HI restriction site (Table 2). A denaturation/renaturation step of the mixed first two PCR products led to an annealing between complementary strands of the common amplified central part, bridging the junction between colicins E3 and E2. This annealed mix was employed as template for the third PCR, performed with the upstream oligo “Nt-ColE3” (used in the 1st PCR), and the downstream oligo “Ct-ImmE2” (used in the 2nd PCR), and led to the amplification of a linear double-stranded hybrid colicin DNA, which was inserted into pACDuet after *Nde*I-*Bam*HI digestion, to obtain plasmid pColE3/ColE2 (Table 1). The same protocol was used to create the colicin E2/colicin E3-ImmE3 chimeric variant fused at two neighbouring residues, G456 of colicin E2 and K455 of colicin E3. The following pairs of oligonucleotides were used successively for this set of three PCR amplifications: “Nt-ColE2” with “ColE2-G456”, “ColE2-ColE3” with “Ct-ImmE3”, and “Nt-ColE2” with “Ct-ImmE3” (Table 2); by employing plasmids pColE2I and pColE3I as templates, successively for the first two PCR steps (Table 1).

Plasmids carrying a hybrid-construction were subsequently transformed into BL21(DE3)-competent cells, prepared in the presence of 10 mM RbCl. The hybrid colicins were coexpressed with Imm proteins, corresponding to the catalytic domains present in the chimeric constructions. Colicin hybrids were produced with the same efficiency as wild-type colicins E2-ImmE2 or E3-ImmE3 and were purified, as wild-type colicins complexes (see above).

Using as template the plasmids carrying wild-type or hybrid colicin derivatives, point mutations, amino acid deletions or additions were generated in the wild-type or hybrid structural *cea* genes by QuikChange site-directed mutagenesis method (Stratagene). Two oligonucleotide primers, containing the desired mutation, each complementary to opposite strands of the plasmid were used to generate mutant derivatives.

### N-terminal Residue Determination of the *in vitro* or *in vivo* Obtained Colicin E2 Forms

The 15 kDa *in vitro* cleaved small colicin E2 form (SC) was transferred from 15% SDS-PAGE to a Problott membrane (Applied Biosystems) for N-terminal peptide sequencing with an ABI 473A automatic sequencer at Institut Pasteur, Paris. The *in vitro* cleavage site between LC and SC forms of colicin E2 was confirmed by mass spectrometric analysis, following digestion by the endoproteinase trypsin, performed on a MALDI-TOF-MS Voyager System 4106 at ESPCI (Paris). The 18.5 kDa processed form (PF) and 33 kDa membrane-associated form (MAF) of colicin E2 were purified by immunoprecipitation, separated by 15% SDS-PAGE and then analysed by mass spectrometric analysis, following digestion by the endoproteinase trypsin or AspN performed at the Institut Curie (Paris).

### Immuno Detection of Processed Colicin E2 (or Colicin E7) Peptides (PF) in the *S100*-cytoplasmic Fraction of Colicin-treated Cells

50 ml cultures of wild-type or mutant *E. coli* strains were grown in LB to OD_600_ = 0.4, at 37°C, then 1 mg of colicin E-ImmE complex was added to the cultures for 45 min. The cells were harvested, washed three-time, resuspended in phosphate buffer pH 7.4 (PBS, Euromedex), and treated with proteinase K at 100 µg/ml for 1 hour at 37°C, to hydrolyse residual colicin molecules bound to the cell surface. After four additional washings the colicin- and proteinase K-treated cells were resuspended in 2 ml PBS buffer with a protease inhibitor complex (Complete EDTA-free; Roche) and then disrupted by sonication. Ribosomes of the soluble fraction were eliminated by micro-ultracentrifugation at 100 S (Beckman rotor TLA 100.2). Proteins of the *S100* cytoplasmic fraction (extract) were separated on 15% SDS-PAGE and analysed by Western blotting with anti-colicin E2-ImmE2 (or E3-ImmE3 or E7-ImmE7) antiserum and detected by ECL chemiluminescence (BIO-RAD, Immun-Star) associated with a CCD camera (BIO-RAD ChemiDoc XR System+). When necessary, ECL detected proteins were quantified by ImageLab software (BIO-RAD).

### Membrane Fractionation of DNase Colicin Treated Target Cells and Immuno Detection of Membrane-associated Colicin E2 (or Colicin E7) Peptides (MAF)

Outer membrane (OM) and inner membrane (IM) fractions were purified from 200 ml non-treated or colicin E-ImmE treated culture (OD_600_ ∼ 0.8) of OmpT-deficient *E. coli* strain AD202. Pelleted and PBS washed cells were resuspended in 7 ml lysis buffer (Tris 10 mM pH 7.5, 10% sucrose, EDTA 1 mM, lysozyme 1 mg/ml, RNaseA 15 µg/ml, DNaseI 50 µg/ml, anti-protease cocktail). Bacteria were broken by two passages through a French Press (1200 psi) and then intact cell and debris were removed by centrifugation (JA20, 8000 rpm, 10 min at 4°C). A partially purified OM fraction was pelleted from the supernatant by ultracentrifugation (72,000 g for 3 min in TLA 100.3 rotor at 4°C). The partially purified IM fraction was pelleted after a second ultracentrifugation of the supernatant (100,000 g in TLA 100.3 rotor for 2 hours at 4°C). OM and IM pellets were resuspended separately in 500 µl storage buffer (Tris 10 mM pH 7.5, EDTA 5 mM) and then layered onto discontinuous sucrose gradients, formed by 0.7 ml of 70% sucrose and 2.3 ml of 50% sucrose [22]. After 20 h of ultracentrifugation (e.g. 192,000 g in TLA 100.3 rotor at 4°C) the isolated OM fraction was collected at the 50–70% sucrose interface and the isolated IM fraction was concentrated at the 20–40% sucrose interface. Membrane fractions (resuspended in storage buffer in the case of OM) were dialyzed against storage buffer. Proteins of membrane fractions were separated by SDS PAGE and the colicin forms were analysed by Western blotting, as described above.

### Isolation of Periplasmic Proteins of DNase Colicin Treated Target Cells

Periplasmic cell extract (PE) was purified from 25 ml culture (OD_600_ ∼0.9) of DNase- colicin treated *E. coli* cells, by a mild osmotic shock procedure. Pelleted and PBS washed bacteria after their treatment by colicin, were resuspended in 1.5 ml TSE buffer (Tris/HCl 20 mM pH 8.0, 20% sucrose, EDTA 1 mM) and then incubated at 4°C for 30 min. Cells were removed by centrifugation in a microtube for 3 min. After precipitation of the supernatant fraction (PE) with acetone, proteins were separated on SDS-PAGE and the colicin forms were analysed by Western blotting, as described above.

## Results

### Colicin Forms Detected in DNase Colicin Treated Bacteria

**Figure 1:**
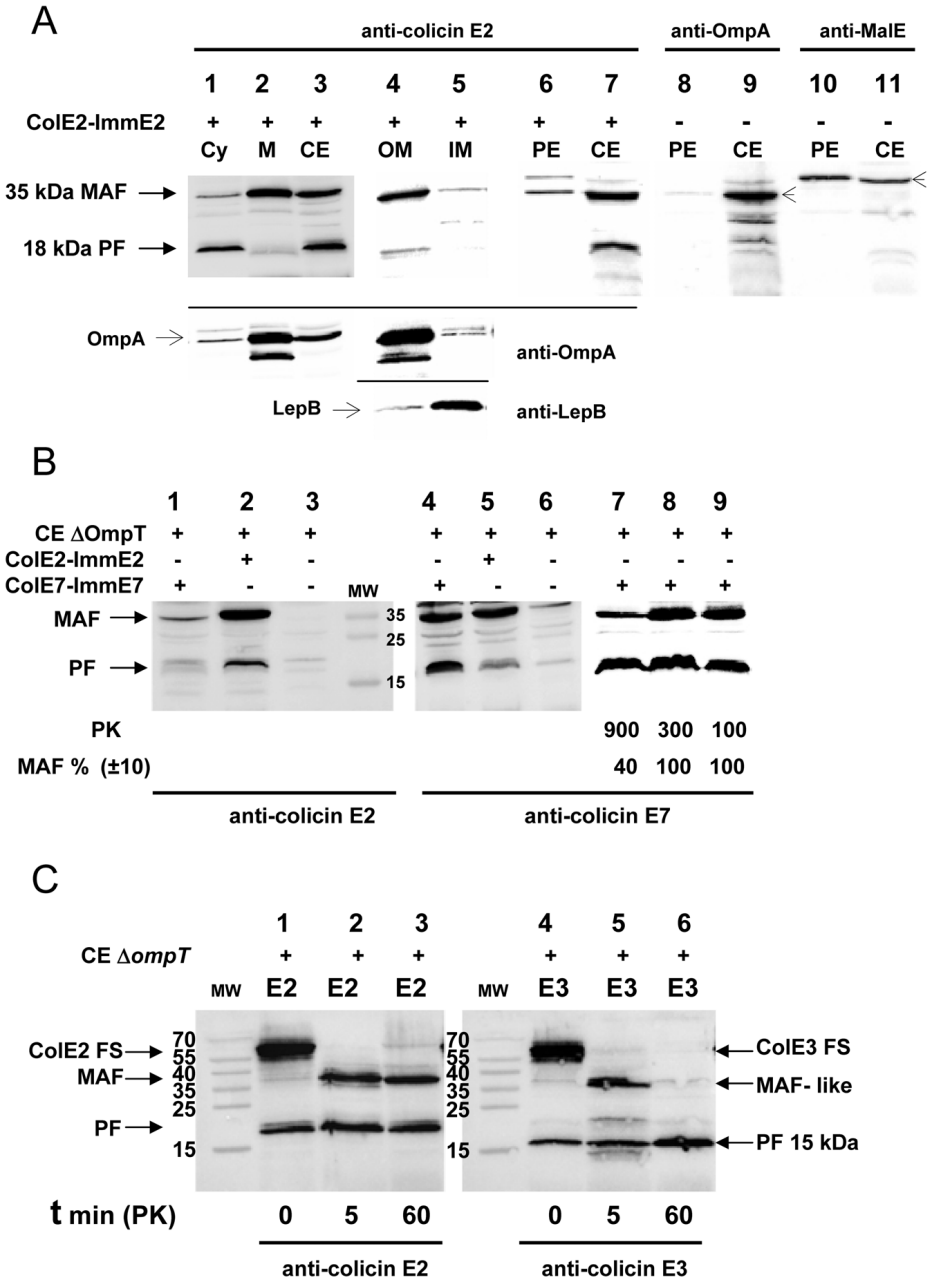
Detection and analysis of the *in vivo* cleaved forms of DNase colicins E2 and E7. (A) The proteins of the crude cell extracts, (*lanes 3, 7, 9, 11,* CE), total membrane (*lane 2*, M), outer membrane (*lane 4*, OM), inner membrane (*lane 5*, IM) and the *S100* cytoplasmic (*lanes 1*, Cy) fractions, isolated from colicin E2-ImmE2 treated (*lanes 1–7,* **+**) or from non treated (*lanes 8–11,* -) cells of an *ompT*-inactivated strain AD202, were separated on 15% SDS-PAGE and analysed by Western blotting with anti-colicin E2 antiserum (*lanes 1–7,* upper part of the panel). The membrane attached form (MAF) and the processed form (PF) with MW of about 35 and 18 kDa, respectively were revealed by ECL. Western blotting with OM specific anti-OmpA (*lanes 1–5*, OmpA: 35 kDa and a smaller degraded form) or with IM specific anti-LepB (*lanes 4, 5*, LepB: 36 kDa) antiserums showed (lower parts of the panel) that the membrane fractionation was efficient. Periplasmic extracts (*lanes 6, 8, 10*, PE) were also analysed with anti-colicin E2 (*lanes 6, 7*) and anti-OmpA (*lanes 8, 9*, OmpA: including some degraded OmpA forms) or anti-MalE that detects the periplasmic maltose-binding protein (*lanes 10, 11,* MBP: ∼43 kDa) antiserums. Proteinase K (PK) treatment (100 µg/ml; 1 h) is systematically applied to colicin-treated cells. (B) CE of OmpT-deficient cells not treated (*lanes 3, 6*) or treated with colicin E7-ImmE7 (*lanes 1, 4, 7–9*) or with colicin E2-ImmE2 (*lanes 2, 5*) were analysed in parallel by Western blotting with anti-colicin E2 or anti-colicin E7 antiserum. PK concentrations (µg/ml) are only indicated when higher concentrations of PK were compared to standard PK concentration of 100 µg/ml (*lanes 7–9*) and some MAF levels were quantified and expressed as a percentage of MAF value, measured with the standard PK concentration (*lane 9*, 100%). The molecular weights (MW) are given in kDa. (C) Identification of a MAF-like peptide from colicin E3-ImmE3 (*lanes 5, 6*, E3) treated bacteria after a 12-fold reduction in the time of hydrolysis (t in min) with PK, in comparison with the endoproteolytic cleavage profile detected from colicin E2-ImmE2 treated cells (*lanes 2, 3*, E2). Analysis of colicin-treated bacteria without PK hydrolysis is shown (t = 0, lanes 1, 4). Proteins of crude cell extracts were separated and analysed by Western blotting with anti-colicin E2 or anti-colicin E3 antiserum.

In the case of RNase colicins an endoproteolytic cleavage step, occurring either before or during translocation across the inner membrane, was shown to be necessary to promote the release of the C-terminal toxic domain into the cytoplasm [10]. To verify that processing of DNase colicins also occurred and was required for import and toxicity, we attempted to detect immunologically any processed colicin forms in the cytoplasm of DNase-colicin treated bacteria. To avoid any interference with the OmpT-dependent colicin cleavage that occurs during colicin contact with the cell surface ([23]–[25] and see below), we used an OmpT-deficient bacteria (strain AD202) for these experiments. We detected in crude cell extracts a *small processed form* (PF) of about 18 kDa (Figure 1A, lane 3) that, as shown below, corresponds to the DNase domain of colicin E2. We localized the PF in the cytoplasmic fraction (Figure 1A, lane 1) similarly to the PF observed in RNase-colicin treated cells. Unlike in the case of the RNase colicins, we also detected a second form of colicin E2 with an apparent MW of about 35 kDa. The second form was found specially enriched in the total membrane fraction, so that it was named *membrane attached form* (MAF) (Figure 1A, lane 2). Further fractionation allowed the separation of the outer and inner membranes, as shown by the specific localization of the OM protein OmpA and the IM peptidase LepB, respectively (Figure 1A, lanes 1–5). A high level of MAF was detected in the outer-membrane, but not in the inner-membrane fraction (Figure 1A, lanes 4, 5). No colicin E2 PF and only traces of colicin E2 MAF were detected in the periplasmic fraction prepared from colicin-treated bacteria (Figure 1A, lane 6). We checked both the enrichment of periplasm-specific maltose-binding protein (MBP), and the absence of OM specific OmpA in our periplasmic extracts (Figure 1A, lanes 8–11).

Colicins E2 and E7 carry 76% identical residues, so that they are considered as closely related antibacterial DNase colicins [26]. Similar MAF and PF forms were also detected with DNase colicin, E7, treated OmpT-deficient cells. They migrated to identical positions on 15% SDS PAGE as the colicin E2 derived MAF and PF (Figure 1B, lanes 2, 4). A partial cross-reaction was observed with the anti-colicin E2 and anti-colicin E7 antiserums (Figure 1B, lanes 1, 5), which can be explained by their protein sequence similarity.

The detection of MAF on cells treated with DNase colicins and the absence of this form with RNase colicins [10], [11] posed the question of whether MAF was a specific DNase colicin intermediate. It should be emphasized that the detection of MAF attached to the OM shows that it is resistant to the proteinase K treatment (PK, 100 µg/ml; 1 h) systematically applied to colicin-treated cells (see *Material and Methods*) (Figures 1A and 1B, lanes 1–6). To investigate the resistance of MAF to proteinase K treatment, colicin E7 treated cells were incubated with 3 fold and 9 fold higher concentrations of proteinase K. There was no decrease in MAF level with 3-fold higher PK, but it decreased 60% with 9-fold higher PK concentration (Figure 1B, lanes 7–9). As expected the level of cytoplasmic PF was not affected, even by the highest PK concentration. Although colicin E2 (or E7) associated with the cell surface becomes resistant to PK, as shown by the detection of MAF, attempts to generate MAF *in vitro* by proteinase K treatment of colicin E2 in complex with its ImmE2 or freed of the Imm protein were unsuccessful. The colicins were completely degraded (Figure S1, lanes 2, 5).

Both DNase E2 and RNase E3 colicins interact with the BtuB receptor via the coiled coil structure of their R-domains. The measured affinities are similar (Kd = 1–2 nM), but the structure is longer in colicin E2 [16], [27]. We wondered, whether a MAF-like peptide could be detected in the case of colicin E3, if we used a milder treatment with PK or if a full-size colicin could be detected on the cell surface in the absence of PK treatment. In fact, without PK treatment a membrane-attached form corresponding to the full-size colicin E2 (FS, 581 amino acids) or E3 was observed (Figure 1C, lanes 1, 4). Astoundingly, after just 5 min of incubation with PK, the full-size colicin E2 or E3 molecules attached to the cell surface had been hydrolysed and generated a MAF or MAF-like peptide, respectively (Figure 1C, lanes 2, 5). When the PK treatment was prolonged for 60 min, all the colicin E3 MAF-like peptide had disappeared, while the colicin E2 MAF was still detectable (Figure 1C, lanes 3, 6). This indicates that MAF is not a physiologically relevant structure *per se*, unlike PF generated during colicin import, but is the result of the proteinase K treatment of the nuclease colicin treated cells. Thus, MAF presumably represents a remnant of the whole nuclease colicin bound to the cell surface. To confirm this interpretation, we checked the presence of MAF on colicin treated cells after removal of free colicins from the medium and a 3 h long incubation preceding the PK treatment. We observed that the level of MAF found was similar to that obtained under our standard conditions (data not shown). Thus the detection of MAF reflects the stable nature of the BtuB-bound DNase colicin complex (see next paragraph). The fact that the T and the major part of R domains (up to position 426) are perfectly conserved between DNase colicin E2 and RNase colicin E3 means that the detection of a stable MAF with DNase colicins is dependent upon the specific C-terminal part of their R-domain and/or the DNase domain, covering together amino acids from position 427 to 581.

### Membrane Components Required for the Processing of DNase Colicins

**Figure 2:**
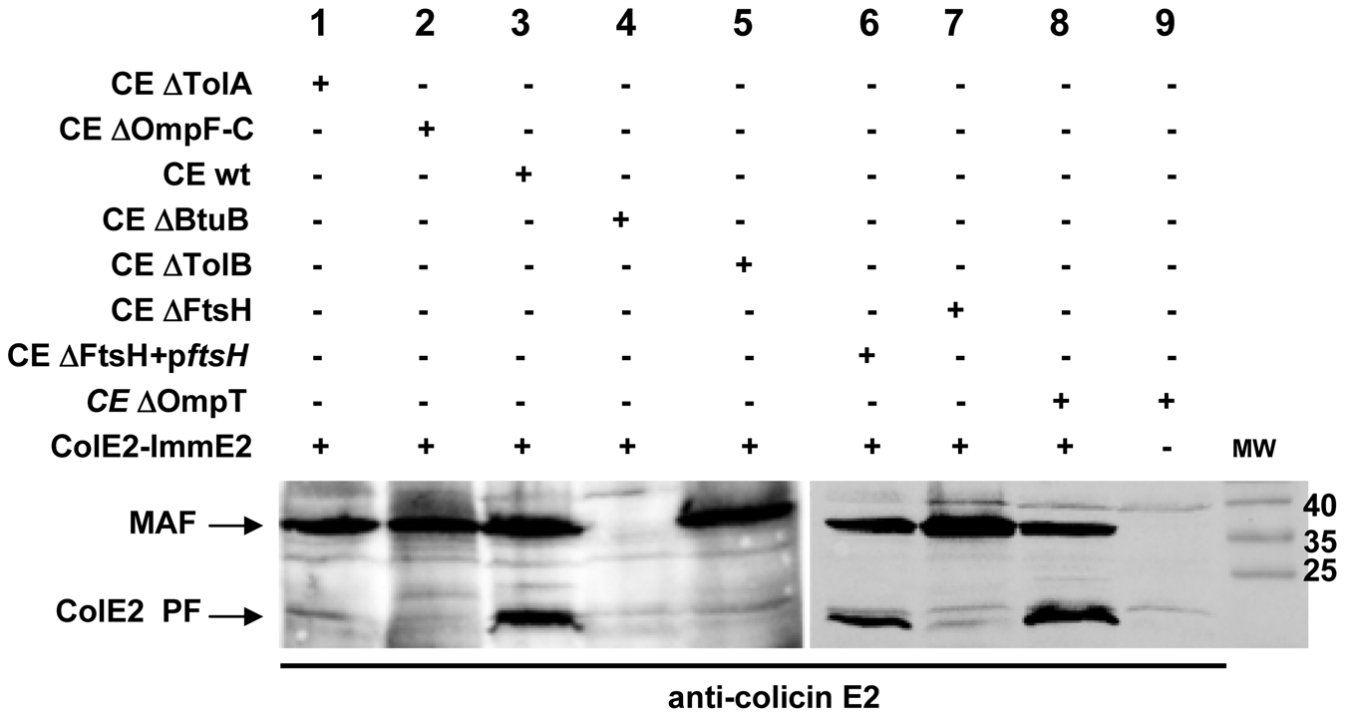
Processing of colicin E2 in membrane-protein deficient strains. The proteins of the crude cell extracts (CE) of wild type (*lane 3*, wt) TolA-deficient (*lane 1*, ΔTolA), porins OmpF and OmpC-deficient (*lane 2*, ΔOmpF-C), BtuB receptor-deficient (*lane 4*, ΔBtuB) and TolB-deficient (*lane 5*, ΔTolB), FtsH protease-deficient (*lane 7,* ΔFtsH) and carrying a plasmid-born wt *ftsH gene (lane 6,* p*ftsH)* and OmpT-deficient (*lanes 8, 9,* ΔOmpT) strains grown in LB medium and treated with ColE2-ImmE2, were loaded onto 15% SDS-PAGE and analyzed by immunodetection with anti-colicin E2 antiserum.

We wondered whether proteolytic processing of DNase colicins was dependent on the membrane proteins already known for their role in the import and toxicity of nuclease colicins. We looked for the presence of the PF, during the penetration of colicin E2 into cells of strains deficient in various membrane proteins. The colicin E2 PF was not detected by western blotting in crude cell extracts of *tolA-*, *ompFC-*, *btuB- or tolB-*inactivated strains (Figure 2, lanes 1–5) in agreement with the involvement of the corresponding membrane components in different steps of the translocation of nuclease colicins across the outer membrane [2]. PF appeared during colicin import only after the translocation of colicin E2 into the periplasmic space, as in the case of RNase colicins. The essential inner membrane FtsH protease is required for sensitivity to both RNase and DNase colicins [13]. In an FtsH-deficient strain no colicin E2 PF was detected, but the processing was fully restored after complementation of the mutant strain with a plasmid-borne wild-type *ftsH* gene (Figure 2, lanes 6, 7). The loss of FtsH prevented the processing and/or translocation of the processed form of colicin E2 into the cytoplasm. The detection of another DNase colicin E7 PF in the cytoplasm of target cells was shown to be dependent on the same membrane components, as in the case of colicin E2, in particular processing to the PF form required FtsH (data not shown).

MAF was regularly detected in the mutant strains with the exception of the BtuB-deficient strain (Figure 2, lane 4), consistent with the hypothesis that it is the binding to BtuB, which protects the colicins from PK. As expected colicin translocation into target cells is not necessary for the detection of MAF, as shown by its presence in an OmpFC-deficient strain. Therefore, the BtuB-attached MAF is present on the extracellular surface of the OM and its detection after treatment by PK is independent of colicin import. These results demonstrated that PF and MAF are generated independently. It should be emphasised that MAF is not a cleavage product produced at the cell surface by OmpT [23], [24], as judged by its presence detected in cell extracts prepared from PK treated OmpT-deficient cells (Figures 1 and 2, lane 8).

### Identification of the PF and MAF of Colicin E2 and Localisation of their N Termini

**Figure 3:**
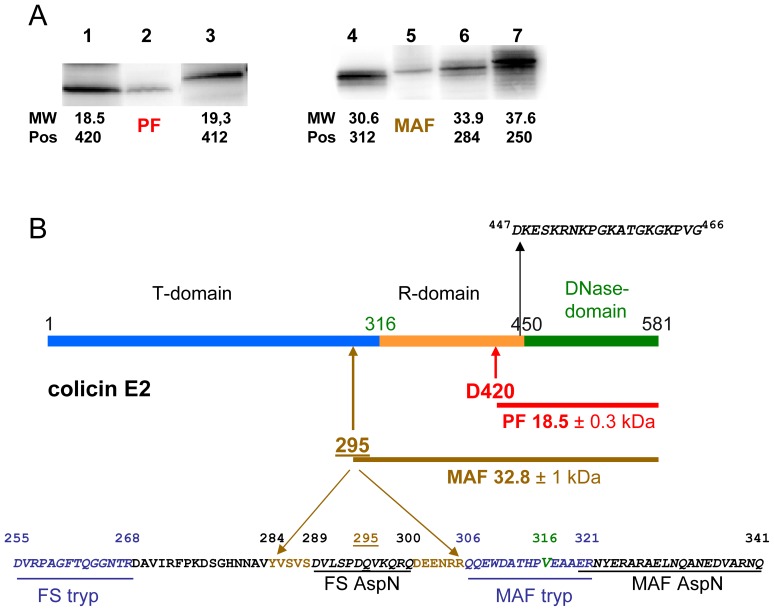
Identification of *in vivo* cleavage products of colicin E2. (A) Comparison of the migration of the *in vivo* processed (*lane 2*, PF) and the membrane attached (*lane 5*, MAF) colicin-E2 forms with various C-terminal peptides of colicin E2, synthesized *in vitro* by a coupled transcription-translation Zubay-30S system. Peptides Asp420-Lys581 (*lane 1*, 18.5 kDa), Asp412-Lys581 (*lane 3*, 19.3 kDa), Ala312-Lys581 (*lane 4*, 30.6 kDa), Tyr284-Lys581 (*lane 6*, 33.9 kDa) and Asn250-Lys581 (*lane 7*, 37.6 kDa) were separated on 15% SDS-PAGE and analyzed by Western blotting with anti-colicin E2 antiserum. (B) The domain structure of colicin E2 is given. Peptide sequence around the junction of the T- and R-domains of colicin E2 (at position V316; indicated in green) is shown. The sequence of the most N-terminal peptide (between positions 447–466) detected in the PF by mass spectrometry (MS) is shown and both the deduced processing site, at or close to residue D420, and size of the PF are indicated. The approximate location of the N-terminal residue of MAF is between positions 284 and 306 (indicated at position 295+/−10 amino acids), as estimated from the migration (A) and mass spectrometric analysis of both the full-size colicin E2 and the colicin E2 MAF, shown at the bottom. Peptides (underlined amino acids in italics) recovered from MS analysis after trypsin (tryp; shown in blue) or AspN (shown in black) treatment are delineated. Peptides, only recovered from the full-size colicin E2 molecule are labelled *FS* while those recovered from both MAF and FS are labelled as *MAF*.

**Figure 4:**
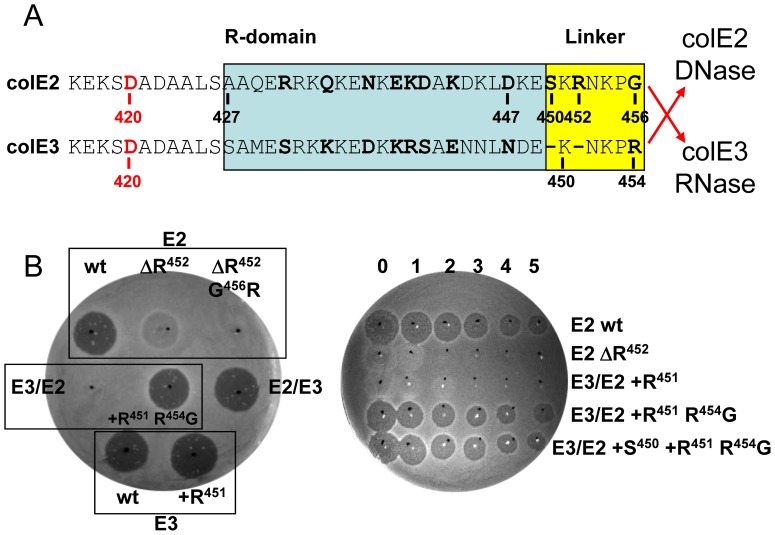
Toxicity of wild-type, mutant and hybrid-colicins E3/E2 and E2/E3 derivatives. (A) Alignment of the C-terminal ends of the R domain (blue box) and the linker regions (yellow box) [15], [16] of colE2 and E3. Amino acids that are specific for either DNase or RNase E-type colicins are shown in bold. Hybrid E2 and E3 colicins were created by in-frame fusion between the upstream R domains and linkers and the downstream nuclease domains as indicated by red arrows. FtsH-dependent processing sites in colicins E2 and E3 at or close to residue D420 (amino acid in red) are shown. (B) Comparison of the cytotoxicity of wild-type (wt; E2 or E3), mutant and hybrid colicins (E3/E2 or E2/E3) by growth inhibition test. Mutant derivatives of the wild-type or hybrids are grouped with their parental strain (black rectangles). R452 was deleted in E2 (ΔR) and was combined with the G456R substitution. In E3/E2 hybrid an R added at position 451 (+R) was combined with the substitution R454G and an S added at position 450 (+S). The *in* *vivo* assay when necessary was quantified by spotting aliquots of undiluted colicin (numbered 0) and serial 10-fold dilutions up to 10^−5^ (numbered 1 to 5) directly onto a lawn of OmpT-deficient AD202 strain (right panel). Dark halos indicate a clear zone of growth inhibition (toxicity), and no clearing indicates resistance to colicin. Turbid zones indicate marginal toxicity.

The *in vivo* detected colicin E2 PF has a size of 18.5 (+/−0.3) kDa, as estimated from comparison with the migration of two *in vitro* synthesized C-terminal peptides of colicin E2, overlapping the catalytic domain (Figure 3A, left panel). This allows us to deduce that the cleavage site that liberates PF is close to residue D420 of colicin E2 (Figure 3B). The location of the processing site in DNase colicin E2 is centered on the same residue as the cleavage in RNase colicin E3 at D420 [10] despite the difference in size of colicin E2 and colicin E3 PFs (15 and 18.5 kDa, respectively). The processing site thus is located near to the C-terminus of the central receptor-binding domains (Figure 3B) and close to the end of the fully conserved 426 N-terminal residues of colicins E2 and E3 (Figure 4A). The location of the processing site of E type nuclease colicins is apparently not dependent on either the size or the peptide sequence of the RNase and DNase domains. Mass spectrometric analysis of colicin E2 PF, following its digestion by the endoproteinase AspN, supports this proposal, since the peptides detected are all derived from the C-terminal part of colicin E2 and the most N-terminal peptide detected corresponded to (447)DKESKRNKPGKATGKGKPVG(466) sequence, which covers the N terminus of the colicin E2 DNase domain (Figure 3B).

The localisation of the colicin E2 MAF within the full-size colicin E2 was performed by a comparative mass spectrometric analysis of both the full-size colicin E2 and the MAF peptide. After their digestion by an endoproteinase, we identified the last common peptides recovered from both the C-terminal half of full-size colicin E2 and MAF, as Q306–R321 and E320–Q341, from trypsin and AspN treatment respectively (Figure 3B). Upstream of these positions we located two adjacent peptides which were only recovered from the full-size colicin E2 molecule digested by trypsin and AspN: D255–R268 and D289–Q300, respectively. These results indicated that MAF includes both the central R- and DNase domains of colicin E2 and allowed the localisation of the MAF N-terminus upstream of position Q306 (Figure 3B). The size of MAF was estimated between 30.6 and 33.9 kDa from comparison with the migration of three *in vitro* synthesized large C-terminal peptides of colicin E2, overlapping both the R- and catalytic domains (Figure 3A, right panel). This implies that the MAF N-terminus should be located downstream of position Y284 (33.9 kDa), so that it was approximately located at position 295 (+/−10 amino acids) near to the C-terminus of the T- domain, corresponding to an estimated size of 32.8 (+/−1) kDa (Figure 3B). Unfortunately, the exact determination of PF and MAF N-termini by peptide-sequencing was not successful.

### Identification of OmpT as the Protease Responsible for the Cleavage of DNase Colicin E2 in Periplasmic Extracts

It had been previously reported that incubation of colicin E7-Imm7 with periplasmic extracts liberated a small C-terminal peptide due to a cleavage between positions K446/R447, although it required extreme conditions with incubation times of 8–12 h. Similarly sized fragments of the DNase domain were also detected *in vivo*, so that it was claimed that this cleavage was required for colicin import, [14], [15]. As deduced from peptide sequence comparison of three DNase colicins, the cleavage site in colicin E7 is equivalent to positions K451/R452 in colicin E2 and K452/R453 in colicin E9. In the case of colicin E2, the proteolytic cleavage at R452 was also suggested as physiologically relevant for colicin translocation [16]. Since these N-terminal cleavage sites are quite distinct from the N- termini generated by FtsH-dependent cleavage at or around position D420 in RNase and DNase colicins that we have identified *in vivo*, it was important to identify the protease responsible for the periplasm-dependent cleavage.

Treatment *in vitro* of colicin E2 with *E. coli* periplasmic extracts, prepared from a wild-type strain, resulted in the cleavage of about 50% of the [^35^S]Met-labelled full-size colicin E2 (62 kDa). The cleavage produced both a 47 kDa large-cleaved (LC) (Fig. S2A) and a 15 kDa small-cleaved peptide forms (SC). Colicin E2 in complex with its cognate immunity protein (colicin E2-ImmE2) was not cleaved, like RNase colicins in complex with their immunity proteins [12], [28]. There was no colicin cleavage *in vitro* in the presence of periplasmic extracts isolated from an OmpT-deficient strain. When purified outer-membrane OmpT protease was added to OmpT-depleted periplasmic extracts, cleavage of colicins E2 was restored (Figure S2A). Therefore, this conclusively identifies the OmpT protease as the unique enzyme responsible for the cleavage of DNase colicins with periplasmic extracts. It should be emphasized that it is known that OmpT has no role in the sensitivity of target cells to colicins [10], [25].

Sequencing the N-terminal amino acids of the small 15 kDa cleaved form (SC), derived from colicin E2, established the N-terminal as (R^452^)NKPGKATGK motif (Figure S2B). This allowed us to localize precisely the cleavage site between two basic residues K451 and R452 in agreement with the consensus for OmpT-dependent cleavage sites [29] and conclude that the SC form carries the C-terminal DNase domain of colicin E2. It is noticeable that this cleavage site is the same as that previously found with periplasmic extracts [14], [15] and is therefore irrelevant for the import of DNase colicins. We note that the authentic *in vivo* processed form of colicin E2 (PF) is 3.5 kDa heavier, than the OmpT-cleaved SC form. The difference corresponds to 32 amino acids separating the start of PF from the R452 located in the linker motif (Figures S2B and 4A).

### Killing Activity of Chimeric E-type Colicins Carrying or not Mutations in the Linker Region

Crystallographic data on the whole RNase colicin E3 defined a short linker between the R-domain and the catalytic domain [30]. This 5–7 amino acid long peptide motif was found to be partially conserved in all nuclease colicins and called “linker region” [15], [16]. Mutagenesis and structural analysis showed previously that R453 residue of DNase colicin E9 located in the linker region was required for the nuclease activity by coordinating the hydrogen bond contacts with phosphate groups of the substrate DNA [31], [32]. However, other reports attributed the loss of toxicity due to mutations affecting the equivalent residues R447 in colicin E7 or R452 in colicin E2, to a processing defect. These mutations were claimed to affect the proteolytic processing step of colicin import into target cells, despite the fact that a 10-fold lower endonuclease activity was also observed with the R447A mutant of colicin E7 [15]. The *in vivo* FtsH-dependent processing site we have identified near D420 is upstream of the linker regions (Figures 3B and 4A). However to eliminate the possibility that the linkers of nuclease colicins are involved in colicin import, we have analysed, in an OmpT-deficient strain, a series of mutations in this region for their effect on both toxicity and processing.

The first 426 residues (43.8 kDa) of colicins E2 and E3 (including the whole N-terminal T- and the major part of the central R-domains) are identical. There are 11 specific amino-acid differences between colicins E2 and E3 in the region 427–456, which are conserved in all RNase or DNase colicins. Three of these changes are located in the linker region (S450 and R452 of colicin E2 are absent and the G456 residue is an R in colicin E3) (Figure 4A). Hybrid colicins E2/E3 and E3/E2 were constructed by switching the RNase and DNase domains after position G456 in colicin E2 and at the equivalent position R454 in colicin E3 (Figure 4A).

Deletion of R452 (ΔR452) or the double mutations ΔR452 G456R introduced in colicin E2 led to marginal and no toxicity, respectively (Figure 4B), in agreement with the previous result for colicin E9 [31], [32]. In contrast, the hybrid colicin E2/E3 was equally toxic as the colicin E3, showing that the amino acids of the linker may not necessarily be E3-type for RNase activity (Figure 4B, left panel). On the other hand, the hybrid colicin E3/E2 had completely lost its toxicity, but it was fully restored in the double mutant +R451 R454G (even in the absence of S450) (Figure 4B), confirming the involvement of amino acids R452 and G456, but not S450 of the linker in cell killing. It is noteworthy, that the single addition +R451 into colicin E3/E2 is not sufficient to restore its toxic activity and the addition of S450 into the double mutant +R451 R454G of colicin E3/E2 did not improve its toxicity up to dilution 10^−5^ (Figure 4B, right panel).

The endonuclease activity of some colicin E2 derivatives was studied *in vitro*. We observed that supercoiled plasmid DNA pUC19 was cleaved into open-circular and linear forms with 5–10 ng full-length colicin E2 freed of the ImmE2. In contrast, the cleavage into open-circular form of the same plasmid DNA required 200 - 300 ng of the non-toxic colicin hybrid E3/E2 or the colicin E2 ΔR452 G456R mutant (Figure S3). These results indicate that R452 and G456 residues of colicin E2, located outside the core of the DNase colicin active site [33], play a role in the DNase activity, as previously demonstrated for the equivalent residue R453 of DNase colicin E9 [32]. Thus, the 30–50 fold decrease in the DNase activity we observed with mutated colicin E2 derivatives (Figure S3) is in turn presumably responsible for the loss of their killing activity. As these residues are important for catalysis, they should be considered part of the catalytic domain in the case of DNase colicins, rather than belonging to the previously described linker regions [15], [16]. In contrast, in the case of colicin E3, one or more changes introducing colicin E2-type residues at the three variable positions of the linker had no effect on the toxicity, so that they do not appear to be required for the colicin E3 RNase activity and could belong to a *bona fide* linker region in colicin E3, as initially described by crystallographic studies [30].

### Processing of Chimeric E-type Colicins

**Figure 5:**
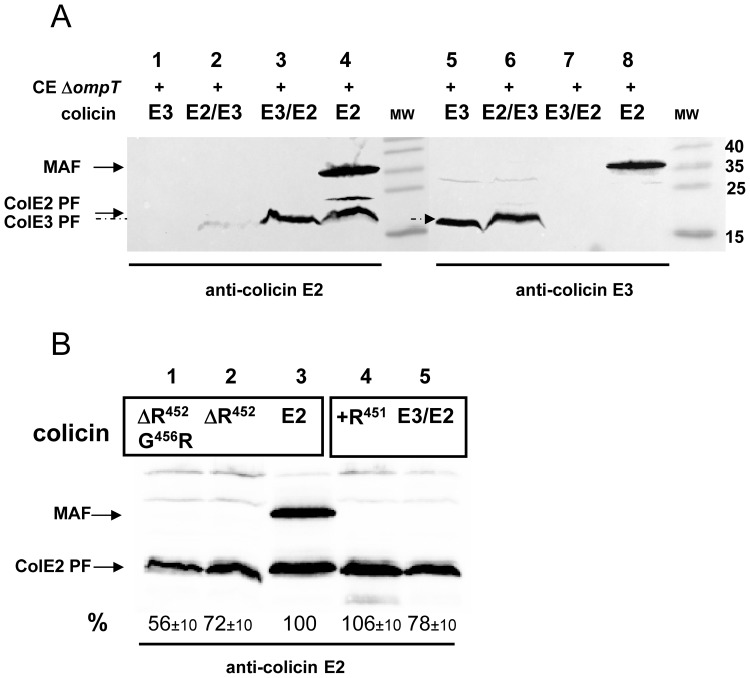
Processing of mutated hybrid colicin E3/2 and E2/E3. (A) Analysis of the *in vivo* observed forms derived from the hybrid-colicins. The proteins of the crude cell extracts of an *ompT*-inactivated strain (AD202; Δ*ompT*), treated by colicin E3 (*lanes 1, 5*, E3), colicin E2 (*lanes 4, 8,* E2), hybrid colicin E2/E3 (*lanes 2, 6*) or hybrid colicin E3/E2 (*lanes 3, 7*), were separated on 15% SDS-PAGE and analysed in parallel by Western blotting with anti-colicin E2 antiserum (left panel) or anti-colicin E3 antiserum (right panel). (B) Colicin forms detected *in vivo* from mutated colicins, were analysed as described in (A). ColE2 PF was quantified and expressed as a percentage of PF value (*%*), measured with colicin E2 (*lane 3*, 100%).

OmpT-deficient bacteria were treated with purified hybrid colicin complexes, E2/E3-ImmE3 or E3/E2-ImmE2 and colicin cleaved forms were detected in the target cells by Western analysis. We observed the presence of colicin E2 processed form in cells treated with colicin E2 or colicin E3/E2 and of colicin E3 PF in cells treated with colicin E3 or colicin E2/E3 (Figure 5A). Processing of the mutated derivatives of colicin E2, ΔR452 and ΔR452 G456R, were to some extent less efficient (56 and 72% of PF), compared to the wild-type E2 (Figure 5B, lanes 1–3). Similarly, colicin E3/E2 with or without the +R451 mutation, exhibited a high level of processing (Figure 5B, lanes 4, 5). It should be highlighted that all non-toxic or only marginally toxic derivatives we constructed (Figure 4B) exhibited a high level of PF formation, showing that efficient processing and translocation across the inner membrane is not influenced by the loss of catalytic activity of these colicins (Figure 5). Therefore, these results show that two specific residues, R452 and G456, despite being essential for toxicity have no function in processing of colicin E2.

A MAF peptide, observed with DNase colicins E2 and E7 (Figure 1B), was not detected with either hybrid colicins E2/E3 or E3/E2 (Figure 5A). Thus, a wild-type colicin E2-type linker present in the colicin E2/E3 hybrid is not sufficient for the detection of MAF (Figure 5A, lane 2). Similarly, the triple mutant restoring a colicin E2-type linker to the hybrid colicin E3/E2 was not able to restore the MAF detection (data not shown). However, E2 linker appeared to be important for detection of MAF because, the deletion of Δ452 in colicin E2 prevented any detection of MAF in target cells (Figure 5B, lane 2). These results indicate that the linker region of DNase colicins is necessary, but not sufficient to allow the formation of a PK resistant MAF peptide at the cell surface.

## Discussion

In the present work we have demonstrated, that an endoproteolytic processing step is essential for the import of DNase colicins E2 and E7 into the cell and for subsequent cell killing. We have shown that only the cytotoxic C-terminal, catalytic domains are detected in the cytoplasm of cells exposed to DNase colicins, as in the case of RNase colicins [10]. The same 18.5 kDa processed form was detected in OmpT-deficient cells treated with colicins E2 or E7 (Figure 1). There is a strict correlation between the loss of sensitivity of FtsH-deficient bacteria to DNase colicins, as previously reported [13], and the absence of PF in the same colicin-treated cells (Figure 3B). The ATPase/endopeptidase FtsH of the inner membrane [34] is therefore the best candidate to function as catalytic enzyme required for colicin processing and entry to the cytoplasm.

The *in vivo* processing site is located at or near position 420, as in the case of RNase colicin E3, so that there are 30 residues in the colicin E2 PF (D420–K450) in addition to the DNase domain (Figure 2). These additional amino acids come from the C-terminal part of the long coiled coil structure of the receptor-binding (R) domain [16]. To allow the processing site to reach the inner membrane and the DNase domain to enter the cytoplasm after its processing, the BtuB bound R-domain must unfold during colicin import [27], [35]. If an earlier import step is prevented by a specific mutation affecting the receptor BtuB or the energy-transducer Tol system, the processing of colicin E2 (or E7) does not occur (Figure 2). Since FtsH is known to act on misassembled or damaged membrane proteins, which are preferentially unfolded, [36], [37] the unfolding of the R and/or C terminal domains may allow FtsH to recognise the colicins as substrates. Numerous different degradation pathways have been described so far for FtsH activity and lend support to the idea of FtsH’s catalytic adaptability [38], [39].

Processing in the periplasm was previously considered as being an essential step for nuclease-colicin import [14], [15]. We showed that in fact, the catalytic enzyme of this cleavage is the outer-membrane protease OmpT [40], [41] (Figure S2), although its presence in the periplasmic extracts is unexpected and most probably due to some contamination. The presence of PF in OmpT-deficient cells (Figure 2), together with the well-known sensitivity of these cells to nuclease colicins, excludes any involvement of the OmpT protease in colicin processing and import. Therefore, the periplasmic cleavage of nuclease colicins is not physiologically relevant to colicin import. Moreover, the OmpT-cleavage site located in colicin E2 at R452 (Figure S2) concerns a residue that plays a role in the DNase activity (Figure S3), as previously shown in colicin E9 [31], [32], but has no role in the authentic processing of DNase colicins (Figures 4 and 5). However, OmpT may play a role at the cell surface, as a part of the bacterial defence system to improve the survival of target bacteria exposed to extracellular antimicrobial peptides [23], [24]. For instance, DNase colicin E2 attached to the cell surface is susceptible to proteolytic cleavage by OmpT into a 50 kDa fragment [25], and this has an antagonistic effect on colicin import, as in the case of RNase colicins [10].

The presence of MAF, detected at the cell surface of DNase-colicin treated cells is not necessary for colicin translocation through the outer membrane or the inner membrane (Figure 2). The MAF attached to the outer membrane is detected only after the PK treatment. The PK-resistant MAF structure includes both the receptor binding and DNase domains (Figure 3B). This indicates that in the absence of PK, numerous DNase colicin molecules, which have not been imported or are “waiting” to be imported, are still strongly associated with the receptor BtuB, when the processed and translocated DNase domain (PF) was detected in the cytoplasm. This result is supported by previous observations, notably derived from colicin competition experiments, which suggested that a partial colicin A molecule or the T- and R-domains of colicin E2 remain in contact with BtuB throughout the import process [42]–[44]. The novelty of our findings is the demonstration of the stable colicin association with the outer membrane BtuB, which concerns whole colicin molecules not yet translocated into the periplasm, as judged by the presence of the catalytic domain present in MAF, rather than colicin molecules whose DNase domains have already penetrated inside the target cells, as suggested previously [44]. Such a stable association presumably facilitates the penetration of incoming colicin molecules into target cells. A MAF-like peptide was detected with RNase colicin E3, but only after a very mild PK treatment of colicin E3 treated cells. The existence of similar MAF, derived from either RNase or DNase colicin molecules, is not really surprising, since the overall translocation mechanism across the outer membrane is known to be very similar for E-type nuclease colicins [2], [45], [46]. MAF resistance to PK is presumably due to intrinsic properties of the MAF peptide sequence, including the RNase or DNase domain and amino-acid changes grouped around the C-terminus of the R-domains. Thus, changes in the coiled coil structure of R-domains that binds BtuB is possibly the key element of the selective resistance of the colicins E2 and E3 MAF to PK.

## Supporting Information

Figure S1 **Proteinase K susceptibility of colicin E2-ImmE2 and colicin E2 freed of ImmE2** ***in vitro*** **.** Colicin E2 (10 µg) in complex with ImmE2 (*lanes 1–2*) or colicin E2 freed of its immunity protein (*lanes 4–5*) was incubated with Proteinase K (PK), 250 µg/ml (*lanes 2 and 5*) in Tris 20 mM pH 7.5 for 30 min. Full-size colicin E2 (FS ColE2, 62 kDa) ImmE2 (10 kDa) and degraded colicin forms were separated on 15% SDS-PAGE and the proteins were detected by Coomassie Blue staining. Proteinase K (29 kDa) without colicin is shown (*lane 3*; including some degraded PK forms). Treatment of neither free colicin E2 nor the colicin E2-ImmE2 complex with PK *in vitro* generated the MAF fragment.
(TIF)

Figure S2 **Identification of the protease responsible for the** ***in vitro*** **cleavage of DNase colicin E2 in periplasmic extracts.** (A) [^35^S]-labelled colicin E2 (1.5 µg), freed of its immunity protein (*lanes 1–3*) or in complex with ImmE2 (*lane 4*), was incubated with periplasmic-protein extracts (PE) prepared from *ompT*-inactivated, AD202 (*lanes 1, 2,* ΔOmpT) or wild-type (*lanes 3, 4,* wt) strains, or with periplasmic extracts supplemented with purified OmpT (including LPS, 0.4 µg [47]) (*lane 2*), for 1 h at 37°C, in 20 mM Na phosphate, 5 mM MgSO_4_ (pH 7.0). After precipitation with acetone, the products were separated by 8% SDS-PAGE and detected by phosphorimaging (GE Healthcare Typhoon): full-size colicin E2 (FS ColE2, 62 kDa) and the large-cleaved form (LC form, 47 kDa). DNase colicins are cleaved in periplasmic extracts only in the presence of OmpT either from wild-type extracts or when added back to ΔOmpT extracts. (B) Location of the *in vitro* cleavage site by OmpT in DNase colicins E2. The sequence of the end of the R domain and junction with DNase domain is shown. The boxed peptide sequence motif at positions 450 - 456 corresponds to the linker region [15], [16]. The OmpT-cleavage site, located between residues K451 and R452, and the corresponding LC and SC (small cleaved) colicin forms are indicated. The N-terminal amino acid sequence of the SC form starting at R452 is indicated (bold).
(TIF)

Figure S3 ***In vitro*** **DNase activity assay of colicin E2 and derivatives.** Circular pUC19 plasmid DNA substrate (500 ng) was incubated in 20 mM Tris pH 7.5 at 37°C for 1 h with various amounts (0 to 300 ng) of purified wild-type colicin E2, mutated colicin E2 derivative, ΔR452 G456R, or hybrid colicin E3/E2, all freed of the ImmE protein. Products were separated on 1% agarose gels and stained with ethidium bromide. Supercoiled double stranded DNA (Sc) was cleaved into open-circular (Oc) and linear (L) forms by 5–10 ng of colicin E2, while 200–300 ng of either colicin E2 (ΔR452 G456R) or hybrid colicin E3/E2 were necessary to cleave DNA mostly into Oc form.
(TIF)
